# Mapping of stripe rust resistance QTL in Cappelle–Desprez × PBW343 RIL population effective in northern wheat belt of India

**DOI:** 10.1007/s13205-016-0380-3

**Published:** 2016-02-16

**Authors:** Sushma Kumari Pawar, Davinder Sharma, Joginder Singh Duhan, Mahender Singh Saharan, Ratan Tiwari, Indu Sharma

**Affiliations:** 1 Indian Institute of Wheat and Barley Research, Karnal, 132001 India; 2 Ch. Devi Lal University, Sirsa, Haryana 125055 India

**Keywords:** Stripe rust, QTL mapping, Adult plant resistance, DArT markers

## Abstract

Stripe rust caused by *Puccinia striiformis* f. sp. *tritici* is most important and devastating disease of wheat worldwide, which affects the grain yields, quality and nutrition. To elucidate, the genetic basis of resistance, a mapping population of recombinant inbred lines was developed from a cross between resistant Cappelle–Desprez and susceptible cultivar PBW343 using single-seed descent. Variety PBW343 had been one of the most popular cultivars of North Western Plains Zone, for more than a decade, before succumbing to the stripe rust. Cappelle–Desprez, a source of durable adult plant resistance, has maintained its resistance against stripe rust for a long time in Europe. Map construction and QTL analysis were completed with 1012 polymorphic (DArT and SSR) markers. Screenings for stripe rust disease were carried out in field condition for two consecutive crop seasons (2012–2013 and 2013–2014). Susceptible parent (PBW343) achieved a significant level of disease i.e., 100 % in both the years. In present investigations, resistance in Cappelle–Desprez was found stable and response to the rust ranged from 0 to 1.5 % over the years. The estimated broad-sense heritability (*h* ^2^) of stripe rust rAUDPC in the mapping population was 0.82. The relative area under the disease progress curve data showed continuous distributions, indicating that trait was controlled multigenically. Genomic region identified on chromosome 2D, was located within the short arm, with flanking markers (*Xgwm484*–*Xcfd73*), explained phenotypic variation (PVE) ranged from 13.9 to 31.8 %. The genomic region identified on chromosome 5B was found with the effect of maximum contribution with flanking DArT markers (1376633|F|0–1207571|F|0), PVE ranged from 24 to 27.0 %. This can, therefore, be utilized for marker assisted selection in developing much needed stripe rust resistant lines for the northern wheat belt of India.

## Introduction

Stripe rust caused by *Puccinia striiformis* is one of the major biotic constraints of wheat production in areas where the cool temperature prevails and affects the grain yield, quality and nutrition. Stripe rust is one of the most important diseases that can cause tremendous losses in wheat production worldwide (Stubbs 1988). Stripe rust can cause yield losses up to 10–70 % and in some cases as high as 100 % yield loss, if the infection occurs at a very early stage and continues to the later stage of the plants (Syed et al. 2007). Major wheat producing area in India fall under North Western Plains Zone (NWPZ) along with the strategic area of wheat cultivation under Northern Hills Zone (NHZ). The disease can be controlled by the use of chemicals, although this method is neither cost effective nor environmentally safe (Rosewarne et al. 2013). Other important effective strategy to control disease is the use of resistant cultivars and the deployment of resistance gene in high yielding varieties. Resistance could be either race specific or non-race specific. Race-specific, also called vertical/seedling/non-durable type of resistances often when deployed is effective only for short-time period (4–5 years). Generally, it is overcome by the new races of pathogen because of constant evolution/selection of races in the nature. On the other hand, non-race-specific resistance (horizontal/partial/slow-rusting/durable or adult plant resistances) shows pleiotropic effect is hard to match by the pathogen and can combat the disease for wide range of pathotypes. A number of such type of slow-rusting genes (showing additive effect) need to be pyramided together to achieve effective genetic control against disease progress in the field. Some of the durable/slow-rusting genes namely *Lr34/Yr18, Lr46/Yr29, Lr67/Yr46* and *Sr2/Yr30* have been reported and found effective in providing rust resistance at field level (Rosewarne et al. 2012). Therefore, it is necessary to search for new source(s) of resistance to identify durable adult plant effective stripe rust resistance against the evolving pathotypes in bread wheat.

Various molecular markers have been widely used to tag and map resistance genes in wheat; however, simple sequence repeat (SSR) has emerged as the choice of marker in gene mapping studies. The development of GBS molecular marker technology that leads to an easy genotyping of large complex genome size such as wheat results in production of detailed genetic map. High throughput markers are not well integrated into the maps, which is the major problem regarding its usage. So, with the help of SSR markers it is easy to locate the positions of different marker types (SNP and DArT) and can build consensus maps between different marker systems. Numerous QTLs (over 140) for resistance to stripe rust in wheat have been reported and through mapping of flanking markers on consensus maps, 49 chromosomal regions have been identified (Rosewarne et al. 2013).

Cappelle–Desprez (European cultivar) was first released in France in 1946 (Lupton and Macer 1962; Worland and Law 1986; Bonjean et al. 2001). It contains seedling resistance genes *Yr3a* and *Yr4a* (De Vallavieille-Pope et al. 1990), but presence of APR against stripe rust in this cultivar is less reported. Cappelle–Desprez maintained a high level of resistance so that it is still recognized as a good source of APR in many European countries.

The objective of the present study was to identify genomic regions controlling adult plant resistance (APR) to stripe rust in recombinant inbred line (RIL) population developed from the cross of ‘Cappelle–Desprez’ (resistant) with ‘PBW343’ (susceptible) parents.

## Methods

### Development and evaluation of RILs

This work was conducted at the Indian Institute of Wheat and Barley Research (IIWBR), Karnal (29°41′0″N, 76°59′0″E) during the two consecutive crop seasons 2012–2013 and 2013–2014, under field condition. A mapping population of 88 F_9_ recombinant inbred lines (RILs) was developed from a cross between resistant Cappelle–Desprez (Vilmorin-27/Hybride-du-Joncquois) and susceptible cultivar PBW343 (ND/VG9144//KALBB/3/YCO “S”/4A/EE#S “S”) using single-seed descend method. The population was further advanced up to F_9_ generations through selfing.

### Stripe rust response test

#### Adult plant stripe rust response test

Planting was done in the first week of November in a randomized complete block design (RCBD) with two replications. About 20 seeds from each line were planted in 1 m row with 20 cm space, comprising two rows in each replication. Susceptible check wheat variety ‘Agra local’ was planted after every 9th plot to ascertain the uniformity of infection throughout the field. Urediniospore-water-tween 20 suspension with stripe rust pathotype *46S119* was atomized at Z12–Z14 Zadok secondary growth stage (Zadoks et al. 1974) according to the National Plant protection Standard (Li et al. 1989). Urediniospores were collected from the actively sporulating plants of susceptible varieties maintained in isolation in polyhouse. Disease severity (DS) data were recorded at weekly interval, for four observations. The disease severity for each line was evaluated for the crop season 2012–2014, under field condition according to the modified Cob Scale (Peterson et al. 1948).

### Molecular marker analysis

Genomic DNA was isolated from leaf sample at two leaf stage using DArT protocol (https://www.diversityarrays.com). Genotyping was done on 88 lines, including the parents with 26, 206 Diversity Array Technologies (DArT) markers by Triticarte Pty Ltd. (Canberra, Australia) and 50 SSR markers located specifically on chromosome 2D and 5B chromosomes (Appels 2003). A total of 1012 (962 DArT and 50 SSR) markers found polymorphic between the parents were used to find the linkage groups. The PCR was done on a thermocycler (BioRad, USA S-1000) with protocol consisting of an initial denaturation at 94 °C for 5 min, followed by 35 cycles of denaturation at 94 °C for 1 min; annealing at 50–60 °C (depending on the individual microsatellite primer) for 1 min; and extension at 72 °C for 1 min followed by a 6 min final extension at 72 °C. Amplification products were resolved by electrophoresis on 3 % agarose gels, visualized by ethidium bromide staining, and gel photograph taken by Geldoc system (Syngene Ltd., USA).

### Statistical analysis

Disease severity data were used to calculate the area under the disease progress curve (AUDPC) for each line and the parent according to the formula,

$${\text{AUDPC}} = \sum \left[ {{{\left( {Y_{i} + Y_{(i + 1)} } \right)} \mathord{\left/ {\vphantom {{\left( {Y_{i} + Y_{(i + 1)} } \right)} 2}} \right. \kern-0pt} 2}} \right]\left( {t_{(i + 1)} - t_{i} } \right),$$

where *Y*_*i*_ = disease severity value on date *t*_*i*_, *t*_(*i*+1)_ − *t*_*i*_ = time (days) between two disease scores *n* = number of times when disease was recorded (Duveiller et al. 1998; Joshi and Chand 2002). The relative AUDPC values were used for the subsequent analysis of variance (ANOVA) and QTL analysis (Lin and Chen 2007). ANOVA was performed using the SAS^®^ 9.3 statistical package (SAS Institute, Cary, NC, USA).

Broad-sense heretability (*h* ^2^) of phenotypic trait (rAUDPC) was calculated using the formula

$$h^{2} = \sigma_{\rm G}^{2} /\left[ {\left( {\sigma_{\rm G}^{2} + \sigma_{\rm GE}^{2} /e + \sigma_{\rm E}^{2} / (e \, \times \, r )} \right)} \right],$$

where *σ*_G_^2^_,_ *σ*_GE_^2^ and *σ*_E_^2^ are estimates of genotypic, genotypic × environment interaction and error variances, respectively, and *e* and *r* are the numbers of environments and replications per environment, respectively (Yang et al. 2005).

Chi test for goodness-of-fit to the expected 1:1 was analyzed for the segregation of individual markers. Polymorphic marker loci exhibiting significant distortion from these expected 1:1 segregation ratios were discarded from the linkage analysis. Chi-square analysis of observed against expected frequencies for disease severity classes was calculated with the “Chi test” function in Microsoft office 2007 Excel. *χ* ^2^ = ∑ (Observed value − Expected value)^2^/(Expected value), where degree of freedom (*df*) = *n* − 1.

**Fig. 1:**
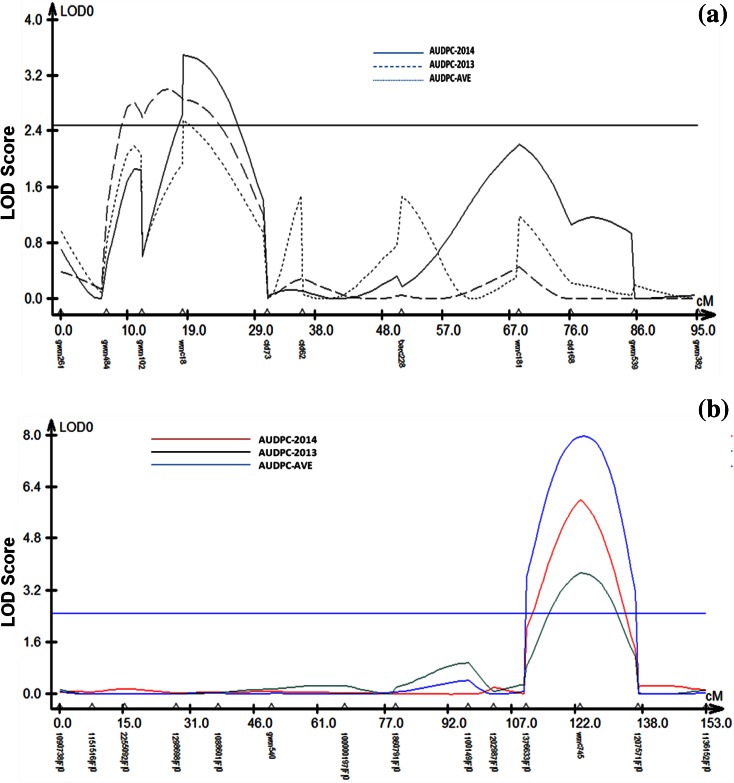
Composite interval mapping of stripe rust severity at adult plant stage in the Cappelle–Desprez/PBW343 mapping population based on stripe rust screening for 2014 and 2013. QTLs were detected on chromosome 2D (**a**) and 5B (**b**) with significant LOD scores

Genetic linkage groups were performed using “MapDisto” Version 1.7 beta software (http://mapdisto.free.fr; Lorieux 2007). Further, grouped markers position was estimated using Join map 4.1 (Van Ooijen 2006). QTL analysis was performed using polymorphic markers falling in a particular linkage group and rAUDPC of the RIL population using composite interval mapping (CIM) with WinQTL Cart V2.5 (Wang et al. 2011). For CIM, significant logarithm of odds (LOD) thresholds was estimated by conducting a permutation test with 1000 interactions at *P* = 0.05 (Fig. 1).

## Results

### Field assessment of stripe rust resistance

**Fig. 2:**
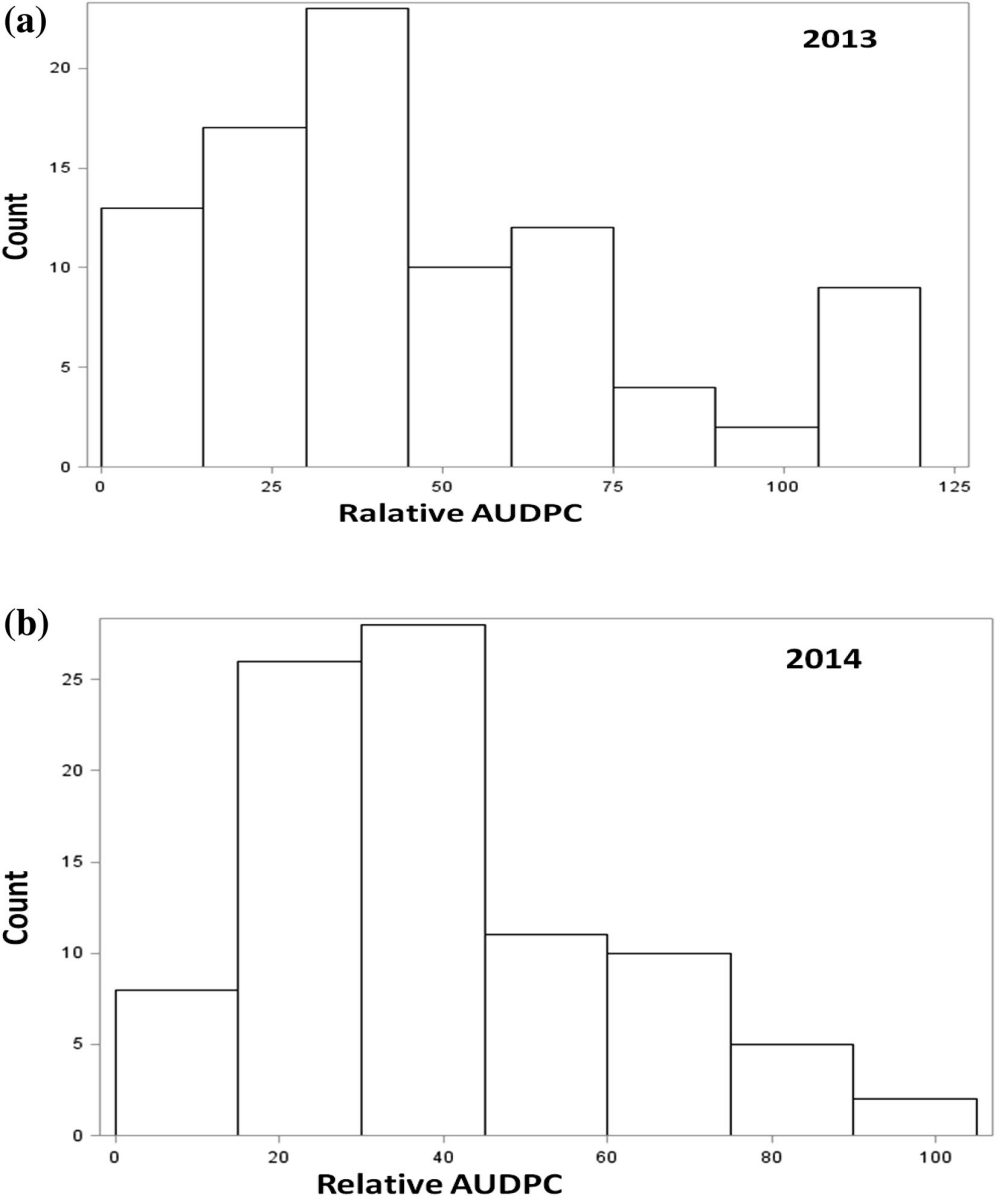
Frequency distribution of relative area under the disease progress curve (rAUDPC) for 88 F_9_ recombinant inbred lines (RILs) for adult plant resistance to stripe rust for year 2013 and 2014

The field studies showed a significance level of disease within the population in both the years (Table 2). Stripe rust severity for the susceptible parent was 100 %; the resistance in Cappelle–Desprez was stable across the years and response to the rust ranged from 0 to 1.5 % over the years. Relative area under disease progress curve (rAUDPC) was calculated from the stripe rust scoring for both crop seasons. No significant differences were detected among replications; and RILs were the only significant source of phenotypic variation observed in the study and interaction between genotype × year was significant; therefore, ANOVA was performed separately for both the years (Table 3). Relative AUDPC during 2012–2013 ranged from 0 to 116.5 with an average of 46.1. For the year 2013–2014, the range of rAUDPC was 1.5–98.5 and mean value was 39.1. The value of standard error (SE) was obtained as 1.0 at 5 % LSD value as shown in Table 2. The estimated broad-sense heritability (*h* ^2^) of stripe rust rAUDPC in the mapping population was 0.82. Phenotypic classification for both the years (2012–2013 and 2013–2014) showing transgressive segregation has been given in the Fig. 2a, b.

### Mapping and QTL analysis

A genetic linkage map was constructed using 50 SSR (18.8 %) along with 962 (3.6 %) polymorphic markers between parents Cappelle–Desprez and PBW343. Twenty-six linkage groups were identified which covered almost all the 21 chromosomes of Wheat. Identified these two groups representing chromosome 2D and 5B containing at least 2 or more SSR loci’s on each. Linkage groups 2D and 5B covered a total map distance of 248 cM.

Linkage groups 2D and 5B containing a total of 11 SSR and 2 SSR + 12 DArT markers covered a map distance of 95 and 153 cM, respectively. Identified two linkage groups were used for QTL analysis using rAUDPC data. QTL mapping using CIM method of WinQTL Cart V2.5 was detected QTL for adult plant stripe rust response variation on chromosome 2D and 5B. A LOD score 2.5 was considered as a significant value to detect a QTL.

Subsequent QTL analysis in the mapping population ‘Cappelle–Desprez × PBW343’ led to the identification of two QTLs (*QYr.iiwbr-5B* and *QYr.iiwbr-2D*) was detected separately and also with mean values of rAUDPC for both the years i.e., 2012–2013 and 2013–2014 (Table 1). The LOD value of rAUDPC for the above years ranged from 3.0 to 3.8 & 3.5 to 6.0, respectively while their corresponding phenotypic variation (PVE) ranged from 13.9 to 31.8 and 24.0 to 27.0 %, respectively. *QYr.iiwbr-5B* conferring with maximum contribution to stripe rust resistance was flanked by DArT markers 1376633|F|0–1207571|F|0 peaks located with *XWmc745* marker. A minor QTLeffect showed on the short arm of chromosome 2D (*QYr.iiwbr-2D*) to stripe rust with flanking marker loci *Xgwm484*–*Xcfd73*. The stripe rust resistance QTLs mapped, their chromosomal location, flanking markers and explained phenotypic variance (PVE) based on composite interval mapping is summarized in Table 1.

**Table 1:** Identification of significant (LOD > 2.5) QTL for stripe rust resistance, their chromosomal location, flanking markers and explained phenotypic variance (*R* ^2^) based on composite interval mapping in Cappelle–Desprez × PBW 343 population

Year	QTL	Marker interval	LOD	Interval size (cM)	PVE (%)	Add
2013	*QYr.iiwbr-5B*	1376633|F|0–1207571|F|0	3.8	26.4	31.8	16.6
	*QYr.iiwbr-2D*	*Xgwm484*–*Xcfd73*	3.0	34.7	13.9	12.7
2014	*QYr.iiwbr-5B*	1376633|F|0–1207571|F|0	6.0	26.4	27.0	17.0
	*QYr.iiwbr-2D*	*Xgwm484*–*Xcfd73*	3.5	34.7	24.0	7.0
Average	*QYr.iiwbr-5B*	1376633|F|0–1207571|F|0	8.0	26.4	34.1	20.2
	*QYr.iiwbr-2D*	*Xgwm484*–*Xcfd73*	2.6	34.7	20.1	8.9

Adult plant effective genomic regions for stripe rust were identified during the present study. Chromosome 2D and 5B carried genes which were found effective for stripe rust pathotype prevalent under the field conditions. These genomic regions were found to be effective for Indian isolates of stripe rust. These genomic regions identified in the present study can be exploited for enhancing the stripe rust resistance in the wheat cultivars through marker assisted selection (Tables 2, 3).

**Table 2:** Range, mean, 5 % LSD and standard error for trait stripe rust in the population

Trait	Year	Range	Mean	5 % LSD	Error
AUDPC	2013	0–116.5	46.1	2.8	1.0
	2014	1.5–98.5	39.1

**Table 3:** Variance components of relative area under the disease progress curve of the F_9_ recombinant inbred line population derived from Cappelle–Desprez/PBW343

Source of variation	*df*	Mean square	*F* value	*P* > *F*
2014				
Gen	87	919.01679	12.17	<0.0001
Rep	1	9.33889	0.12	0.7260^NS^
Error		75.54114		
2013				
Gen	87	1975.7017	780.71	<0.0001
Rep	1	0.2722	0.11	0.7437^NS^
Error		2.5306		

## Discussion

Stripe rust severity for the susceptible parent (PBW343) achieved a significant level of disease i.e., 100 % in both the years. In India, PBW343 had been one of the most popular and ruling varieties of North Western Plains Zone (NWPZ). Lines PBW343 and Inqualab 91 along with their derivatives not only carried high levels of resistance to leaf and stripe rusts or both, but also showed about 5–15 % higher yield potential than the original cultivars (Singh et al. 2004). Stripe rust resistance in PBW343 (in India), Inquilab-91 and Bakhtwar (in Pakistan), Chamran and Shiroudi (in Iran), Kubsa (in Ethiopia) and Cham 8 (in Syria) was based on *Yr27*. Breakdown of *Yr27* resistance in PBW343, Inquilab 91 and Chamran, in India, Pakistan and Iran, respectively, was reported between 2002 and 2004. Although occasional stripe rust outbreaks appeared in some other wheat growing areas of the world as well but due to unfavorable environmental conditions increase in the severity of *Yr27* virulent pathotypes got restricted.

In present investigation resistance in Cappelle–Desprez was found stable and response to the rust ranged from 0 to 1.5 % over the years. Cappelle–Desprez (European cultivar) was first released in France and due to its resistance to stripe rust it occupied a large acreage across Western Europe for a very long duration, until the late 1970s (Bonjean et al. 2001). Worland and Law (1986) and Law and Worland (1997) reported high level of APR for number of major diseases, including stripe rust as a main reason of longevity of this cultivar. Due to the longevity of this cultivar, it was extensively used in many breeding programmes such as UK and in many European countries (Angus 2001; Bonjean et al. 2001; Porche 2001). Cappelle–Desprez although known for having seedling resistance genes *Yr3a* and *Yr4a* (De Vallavieille-Pope et al. 1990), also maintained a high level of resistance in many European countries due to its adult plant resistance.

Present study leads to the identification of *QYr.iiwbr-5B* on short arm of chromosome 5B with flanking DArT markers (1376633|F|0–1207571|F|0), peak located with *XWmc745* marker explaining 34.1 % phenotypic variation with mean rAUDPC value. Mallard et al. (2005) identified two QTL (*QYr. inra-5B.1* and *QYr. inra-5B.2*) with SSR loci *Xgwm544* in Camp Remy which mapped to the telomeric region on the long arm of chromosome 5B.

Riley et al. (1967) and Badaeva et al. (2007) reported that Cappelle–Desprez carries the reciprocal, centromeric translocations 5BL–7BL and 5BS–7BS. Its parentage (vilmorin and Hybride du Joncquois) also possesses this translocation (Worland and Law 1986; Law and Worland 1997). Boukhatem et al. (2002), however, in their study, did not identify any QTL on chromosome 5B in Camp Remy. Stripe rust resistance QTL on chromosome 5B have also been detected in the Italian cvs Libellula and Strampelli (Lu et al. 2009), the Israeli cv. Oligoculm (Suenaga et al. 2003), the Australian cv. Janz (Bariana et al. 2010) and the French cv. Flinor (Feng et al. 2011). Feng et al. (2011) identified two QTLs on chromosome 5B which are expressed at the seedling stage and at higher temperatures (*QYr-tem-5B.1* and *QYr-tem-5B.2*) in Flinor. Flinor has ancestral relationship with Cappelle–Desprez and one of these QTL, *QYr-tem-5B.1,* overlaps with the region defining *QYr.ufs-5B*. *QYr-tem-5B.1* explained up to 33 % of the phenotypic variation in Flinor.

Present investigation, also, mapped a minor QTL region on chromosome 2D with significant LOD value. *QYr.iiwbr-2D*, although defined by a smaller marker interval with flanking SSR markers (*Xgwm102*–*Xcfd73*), was found contributing less significantly to the explained phenotypic variance i.e., 20.1 % with rAUDPC mean value of both the years. Mallard et al. (2005) identified a QTL (*QYr.inra-2DS*) near to the centromere on 2DS in the cv. Camp Rémy between marker loci *Xgwm102* and *Xgwm539* explaining 24–69 % phenotypic variance, although the reason for explained low phenotypic variance may be probably due to differences in environmental conditions or influenced by genetic background.

Worland and Law (1986) and Worland et al. (1988) suggested that *Yr16*, gene is located on the centromeric region of chromosome 2D and this was further confirmed through cytogenetic analysis. Devos et al. (1993) and Agenbag et al. (2012) placed the gene 9.3 cM from the centromere between RFLP marker loci *Xpsr641-2D* and *Xpsr681-2D* on a consensus map, Ta-Gale-2D (http://wheat.pw.usda.gov/GG2/index.shtml).

Bariana et al. (2001) reported temperature-sensitive seedling resistance, *YrCK* on chromosome 2D in the Australian cv. Cook and a derivative, cv. Sunco explaining 13–19 % of the phenotypic variance for stripe rust and 9–13 % of the phenotypic variance for leaf rust resistance (Navabi et al. 2005). *QYr.caas-2DS*, explaining 8.4–12.1 % of the phenotypic variance, was identified in the Italian cv. Libellula, (Lu et al. 2009), while Suenaga et al. (2003) detected QTL explaining less than 10 % of the phenotypic variance in the cv. Oligoculm in this region on 2DS. Stripe rust resistance QTL were identified and mapped on the short arm of chromosome 2D, close to the *QYr.ufs-2D* in the UK cvs Guardian (Melichar et al. 2008) and Claire (Powell 2010), both of which have Cappelle–Desprez in their pedigrees.

Present investigation confirms the effectiveness of genomic regions on chromosome 2D and 5B for stripe rust pathotypes in our population in Indian conditions. These may be exploited for enhancing the level of resistance against stripe rust in the cultivated wheat germplasm through marker assisted selection.
